# *Ureaplasma* positivity and respiratory outcomes in very low birth weight infants: a cohort study with a nested double-blind randomized pilot trial of azithromycin

**DOI:** 10.1186/s12887-026-07199-2

**Published:** 2026-07-03

**Authors:** Euiseok Jung, Geun Mi Park, Chae Young Kim, Jeong Min Lee, Ha Na Lee, Jiyoon Jeong, Soo Hyun Kim, Byong Sop Lee, Ellen Ai-Rhan Kim, Ki-Soo Kim

**Affiliations:** 1 Division of Neonatology, Department of Pediatrics, Asan Medical Center Children’s Hospital, University of Ulsan College of Medicine, 88, Olympic-ro 43-gil, Songpa-gu, Seoul, 05505 Korea; 2 Department of Pharmacy, Asan Medical Center, Seoul, Korea

**Keywords:** Azithromycin, Bronchopulmonary dysplasia, Preterm infant, Ureaplasma, Very low birth weight infant

## Abstract

**Background:**

*Ureaplasma* spp. colonization/infection has been implicated in the inflammatory pathogenesis of bronchopulmonary dysplasia (BPD) in very low birth weight (VLBW) infants, but the benefit of targeted macrolide therapy remains uncertain. We evaluated respiratory outcomes associated with early *Ureaplasma* positivity in VLBW infants and assessed feasibility and preliminary efficacy of a standardized intravenous azithromycin regimen in a nested pilot randomized trial.

**Methods:**

This single-center study comprised (1) a retrospective cohort of inborn VLBW infants (< 1,500 g) born between July 2017 and December 2021 who underwent *Ureaplasma* screening within 24 h after birth, and (2) a prospective, double-blind, randomized, placebo-controlled pilot trial nested among *Ureaplasma*-positive infants requiring significant respiratory support. In the cohort, outcomes included moderate-to-severe and severe BPD at 36 weeks’ postmenstrual age and home oxygen therapy. Multivariable logistic regression adjusted for gestational age, sex, preterm premature rupture of membranes, histologic chorioamnionitis, antenatal steroid exposure, patent ductus arteriosus treatment, and sepsis. In the trial, infants were randomized 1:1 to intravenous azithromycin for 14 days (10 mg/kg/day for 7 days, then 5 mg/kg/day for 7 days) or placebo. Microbiologic response at 2 weeks and safety, including QTc, were assessed.

**Results:**

Among 536 VLBW infants, 34 (6.3%) were *Ureaplasma* positive. *Ureaplasma*-positive infants had higher rates of moderate-to-severe BPD (76.5% vs. 41.0%), severe BPD (67.6% vs. 36.0%), and home oxygen therapy (46.2% vs. 19.1%). After adjustment, *Ureaplasma* positivity remained associated with moderate-to-severe BPD (adjusted odds ratio [aOR] 3.86, 95% confidence interval [CI] 1.44–10.35) and severe BPD (aOR 2.93, CI 1.21–7.10). In the pilot trial (*n* = 26; 13 per group), moderate-to-severe BPD occurred in 61.5% (8/13) with azithromycin versus 84.6% (11/13) with placebo, and severe BPD in 46.2% (6/13) versus 76.9% (10/13). Loss of detectable *Ureaplasma* by culture at 2 weeks occurred in 13/13 azithromycin-treated infants, while 5/13 placebo infants remained positive. No QTc prolongation or clinically significant safety concerns attributable to azithromycin were observed.

**Conclusion:**

Early *Ureaplasma* positivity in VLBW infants was associated with substantially worse respiratory outcomes. A two-week intravenous azithromycin regimen was feasible, microbiologically active, and showed directionally favorable estimates for BPD without an observed safety signal. Larger microbiology-guided trials are warranted.

**Trial registration:**

KCT0002373 (registered 7 July 2017).

**Supplementary Information:**

The online version contains supplementary material available at https://doi.org/10.1186/s12887-026-07199-2.

## Background

Bronchopulmonary dysplasia (BPD) remains one of the most consequential morbidities among very preterm and very low birth weight (VLBW) infants, and its prevention has become increasingly important as survival continues to improve with advances in neonatal intensive care [1–3]. BPD is a complex, multifactorial chronic lung disease characterized by disrupted alveolar and vascular development, in which antenatal exposures and postnatal lung injury together contribute to impaired lung growth and long-term respiratory sequelae [1–3]. Despite the widespread adoption of strategies such as optimizing noninvasive respiratory support, minimizing ventilator-induced lung injury, and improving nutrition, clinically meaningful reductions in moderate-to-severe BPD remain challenging, particularly in infants at the limits of viability [1–3].

Inflammation is central to the pathogenesis of BPD [1, 4, 5]. Antenatal inflammation, including chorioamnionitis, has been linked to altered fetal lung development and may prime the immature lung to respond excessively to postnatal insults such as mechanical ventilation, oxygen toxicity, and sepsis [4, 5]. Among organisms implicated in perinatal infection, *Ureaplasma* species have attracted sustained attention because they are frequently encountered in the maternal genital tract and are among the earliest organisms acquired by preterm infants [6, 7]. Experimental and clinical evidence suggests that respiratory tract colonization or infection with *Ureaplasma* can trigger persistent inflammatory responses in the immature lung, potentially contributing to arrested alveolarization and fibrotic remodeling that predispose to BPD [6, 7].

Consistent with this biological rationale, systematic reviews and meta-analyses have reported an association between pulmonary *Ureaplasma* colonization and subsequent BPD, although heterogeneity in study populations, specimen types, and BPD definitions has contributed to ongoing debate regarding causality and the magnitude of effect [8, 9]. Importantly, if *Ureaplasma* is a modifiable contributor to BPD, targeted antimicrobial therapy could offer an actionable prevention strategy beyond supportive care alone. Macrolides are attractive candidates because of their activity against *Ureaplasma* and additional anti-inflammatory properties [7].

However, clinical evidence for macrolide therapy in preterm infants remains mixed, and optimal regimens are not established. A randomized trial of clarithromycin in *Ureaplasma urealyticum*-positive preterm infants reported reduced BPD incidence in the treatment group, but generalizability to other settings and macrolides is uncertain [10]. Trials of azithromycin have suggested potential benefit in selected populations, while pharmacokinetic work supports the importance of achieving adequate early exposure to facilitate microbial clearance [11, 12]. In designing a pragmatic pilot intervention for *Ureaplasma*-positive VLBW infants, we selected a standardized two-week regimen intended to provide sustained early coverage while limiting prolonged macrolide exposure, thereby balancing feasibility and safety in a real-world neonatal intensive care setting.

Against this background, we first evaluated the association between respiratory *Ureaplasma* positivity and respiratory outcomes in a VLBW cohort. We then conducted a double-blind, randomized, placebo-controlled pilot trial to assess the feasibility and preliminary efficacy of a standardized two-week azithromycin regimen among *Ureaplasma*-positive infants, with particular focus on moderate-to-severe BPD and severe BPD outcomes after adjustment for key perinatal confounders.

## Materials and methods

### Study design and setting

This two-part study was conducted at Asan Medical Center Children’s Hospital (Seoul, Republic of Korea) and comprised (1) a retrospective cohort analysis of inborn VLBW infants and (2) a single-center, prospective, randomized, double-blind, placebo-controlled, parallel-group pilot trial nested within the cohort.

### Study population

The cohort included all inborn VLBW infants with birth weight < 1,500 g born between July 2017 and December 2021. Infants with major congenital anomalies were excluded from cohort analyses (chromosomal abnormalities, hydrops fetalis, critical congenital heart disease, congenital diaphragmatic hernia, and major pulmonary anomalies).

### *Ureaplasma* screening and microbiologic methods

All cohort infants underwent *Ureaplasma* screening within 30 min to 24 h after birth. Specimens were obtained from gastric aspirate and, when clinically feasible, respiratory samples (endotracheal aspirate in intubated infants or nasopharyngeal aspirate in non-intubated infants). Detection and identification of *Ureaplasma* spp. were performed by culture using the Mycoplasma IST3 kit (bioMérieux, Marcy-l’Étoile, France) according to the manufacturer’s instructions. Culture results were interpreted and reported by board-certified laboratory medicine physicians in the Department of Laboratory Medicine as part of routine clinical practice. *Ureaplasma* positivity was defined as a positive culture result from any sampled site (gastric aspirate and/or respiratory specimen), without prioritizing one specimen type over another. For sensitivity analyses, respiratory-specimen positivity was defined as culture positivity from endotracheal or nasopharyngeal aspirates (with or without concomitant gastric aspirate positivity), excluding infants with gastric-aspirate-only positivity.

Specimens were collected as follows:

- *Endotracheal aspirate (intubated infants only)*: With the infant supine, 0.5 mL sterile saline was instilled via the endotracheal tube, followed immediately by suction; aspirate was collected into a suction trap.
- *Nasopharyngeal aspirate (non-intubated infants only)*: With the infant supine, 0.5 mL sterile saline was instilled into the nasal cavity, followed immediately by suction; aspirate was collected into a suction trap.
- *Gastric aspirate (all infants)*: With the infant supine, a 5-Fr orogastric tube was inserted orally and gastric aspirate was obtained immediately using a 5 mL syringe.

For trial participants, a follow-up *Ureaplasma* culture was planned within 3 days after completion of study drug to assess microbiologic response.

### Pilot trial eligibility, randomization, and masking

Among cohort infants with culture-confirmed *Ureaplasma* positivity, infants were screened for the nested pilot trial. After written informed consent was obtained from both legal guardians, eligibility was confirmed based on prespecified respiratory criteria at the time of trial enrollment. We included “significant respiratory support” to enrich for infants at higher baseline risk of clinically meaningful BPD outcomes and to improve feasibility of detecting a treatment signal in this pilot trial. “Significant respiratory support” was defined as requiring FiO₂ ≥0.40 and/or invasive or noninvasive respiratory support, including conventional mechanical ventilation, high-frequency oscillatory ventilation, synchronized noninvasive positive pressure ventilation, noninvasive positive pressure ventilation, nasal continuous positive airway pressure, or heated humidified high-flow nasal cannula.

Exclusion criteria included (1) inability to obtain study specimens because of respiratory instability and (2) prolonged corrected QT interval on screening electrocardiography (QTc > 450 ms). Screening electrocardiography was performed after consent and before randomization and study drug initiation. Infants who met all eligibility criteria, including QTc screening, proceeded to randomization, and the assigned study treatment was initiated promptly thereafter.

Eligible infants were randomized 1:1 to azithromycin or placebo using block randomization with stratification by birth weight (< 1,000 g vs. ≥ 1,000 g). The allocation sequence was concealed from investigators. Study drug preparation and dispensing were performed by a pharmacist not involved in clinical care or outcome assessment. Legal guardians, clinicians, investigators, and outcome assessors remained blinded to group assignment throughout the trial.

### Intervention and concomitant medications

Infants assigned to the intervention group received intravenous azithromycin (Zithromax^®^, Pfizer) infused over 1 h once daily for 14 days: 10 mg/kg/dose every 24 h for 7 days followed by 5 mg/kg/dose every 24 h for 7 days. The placebo group received intravenous normal saline in matched volume and identical administration schedule. This 14-day step-down regimen was selected to provide sustained exposure while maintaining feasibility and tolerability in a fragile population. Dosing was weight-based according to the prespecified regimen and was not adjusted by gestational age or postnatal age. No protocolized renal dose adjustment was applied. Study drug administration could be temporarily withheld at the discretion of the clinical team in the setting of clinically significant organ dysfunction. Initiation of the assigned study treatment occurred after culture confirmation of *Ureaplasma* positivity. Timing was primarily determined by the time required to obtain culture results, and could also be influenced by clinical stability and completion of written parental consent.

Standard NICU care, including respiratory support strategies, was provided according to unit-wide protocols in both groups and was not altered by study participation or treatment allocation. Permitted concomitant medications included methylxanthines (aminophylline/theophylline and caffeine citrate), diuretics (furosemide, spironolactone, thiazides), and analgesia/sedation (fentanyl), as clinically indicated. Systemic corticosteroids for evolving BPD were permitted according to unit practice; dexamethasone could be considered in infants who remained ventilator-dependent beyond 7 days of life and were judged to have high predicted risk of death or moderate-to-severe BPD at 36 weeks’ postmenstrual age using the prediction model by Laughon et al. [13]. Other systemic antibiotics could be administered during the treatment period as clinically indicated as part of standard NICU care. Probiotics were not used in our NICU during the study period. Phenytoin was prohibited due to potential drug interactions. Treating clinicians remained blinded to treatment allocation throughout the intervention period.

### Outcomes and definitions

#### Cohort analysis

Maternal and perinatal variables included gestational age, birth weight, sex, multiplicity, maternal hypertension, maternal diabetes, antenatal corticosteroid exposure, mode of delivery, histologic chorioamnionitis, and preterm premature rupture of membranes (PPROM). Maternal comorbidities were based on obstetric records. Antenatal corticosteroid exposure was defined as receipt of at least one dose prior to delivery. Histologic chorioamnionitis was defined as a diagnosis documented in placental pathology reports. Placental histopathologic examination was routinely requested for VLBW deliveries when feasible as part of institutional practice, although a placental pathology report was not available for all infants during the study period. PPROM was defined as rupture of membranes before the onset of labor prior to 37 weeks’ gestation.

Respiratory distress syndrome was defined by typical clinical and radiographic findings requiring respiratory support with or without surfactant therapy. Air leak syndrome included radiographically confirmed pneumothorax, pneumomediastinum, or pulmonary interstitial emphysema. Patent ductus arteriosus (PDA) requiring treatment was defined as receipt of medical therapy and/or surgical or catheter-based closure.

Necrotizing enterocolitis was staged according to Bell’s criteria, and clinically significant NEC was defined as stage ≥ 2 [14]. Systemic postnatal corticosteroid exposure was defined as administration of systemic corticosteroids for evolving BPD as part of routine clinical care. Late-onset sepsis was defined as a positive blood culture obtained after 72 h of life with antibiotic treatment. Intraventricular hemorrhage was classified using the Papile grading system, and severe IVH was defined as grade ≥ 3 [15]. Retinopathy of prematurity was classified according to the International Classification of Retinopathy of Prematurity (ICROP) revisited (2005), and severe ROP was defined as stage ≥ 3 [16]. Bronchopulmonary dysplasia severity was assessed at 36 weeks’ postmenstrual age using the 2001 NICHD consensus definition [3]. For analyses, “moderate-to-severe BPD” was defined as the composite of moderate or severe BPD according to the 2001 NICHD criteria. Home oxygen therapy after discharge was ascertained from outpatient follow-up records and defined as nasal-prong oxygen use at a flow rate of ≥ 0.1 L/min.

### Pilot trial

The primary outcomes were moderate-to-severe BPD and severe BPD at 36 weeks’ postmenstrual age. Secondary outcomes included duration of invasive mechanical ventilation, duration of noninvasive respiratory support, home oxygen therapy, and microbiologic response defined as loss of detectable *Ureaplasma* by culture after completion of study drug.

Safety was monitored throughout therapy using routine NICU cardiopulmonary monitoring, including continuous bedside ECG monitoring. Standard 12-lead ECGs were obtained at enrollment (baseline, prior to randomization and study drug initiation), approximately 1 week after treatment initiation (± 1 day), and within 3 days after completion of the 14-day course to assess QTc, and infants with baseline QTc prolongation (QTc > 450 ms) were excluded. Laboratory monitoring followed routine NICU practice and included complete blood count, renal function and electrolytes, liver enzymes and bilirubin, and inflammatory and infection-related tests (CRP and blood culture). Adverse events were assessed at the prespecified time points and were also recorded whenever clinically suspected during routine care.

Exploratory neurodevelopmental outcomes at 3 years of corrected age were assessed when follow-up evaluations were available using the Bayley Scales of Infant Development, Second Edition (BSID-II). Severe delay was defined as a mental developmental index (MDI) or psychomotor developmental index (PDI) score < 70. Cerebral palsy, visual impairment, and hearing impairment were collected from follow-up clinical assessments documented in outpatient records.

### Handling of missing data and denominators

No imputation was performed. Analyses were conducted using available data for each variable, and denominators therefore varied across outcomes. Placental histopathology (for histologic chorioamnionitis) was not available for all deliveries. BPD outcomes were assessed at 36 weeks’ postmenstrual age among infants who survived to 36 weeks and had documented 36-week respiratory status.

### Statistical analysis

Categorical variables are presented as n (%) and continuous variables as mean (standard deviation) or median (interquartile range), as appropriate.

For descriptive comparisons in the cohort, categorical variables were compared using Fisher’s exact test and continuous variables using the Mann–Whitney U test, as appropriate. Multivariable logistic regression was performed to estimate adjusted odds ratios (aORs) and 95% confidence intervals for BPD outcomes associated with *Ureaplasma* positivity. Prespecified covariates included gestational age, sex, preterm premature rupture of membranes (PPROM), histologic chorioamnionitis, antenatal steroid exposure, PDA treatment, and sepsis. Postnatal respiratory severity variables (e.g., intubation/invasive ventilation) were not included in the primary models because they may be downstream of *Ureaplasma* detection and lie on the causal pathway to BPD. Adjusting for such post-exposure variables could introduce overadjustment bias and attenuate estimates toward the null.

For the pilot trial, between-group comparisons were performed to facilitate interpretation of baseline characteristics and key clinical outcomes. Continuous variables were compared using the Mann–Whitney U test and categorical variables using Fisher’s exact test, as appropriate. P-values were considered exploratory given the pilot sample size and were not adjusted for multiple comparisons. For binary outcomes, risk differences and 95% confidence intervals were calculated using the Newcombe method with Wilson score intervals, and risk ratios and 95% confidence intervals were calculated on the log scale. Accordingly, two-sided P values < 0.05 were interpreted cautiously and in conjunction with effect sizes and confidence intervals. Statistical analyses were performed using IBM SPSS Statistics (version 27; IBM Corp., Armonk, NY, USA) and R (version 4.5.2; R Foundation for Statistical Computing, Vienna, Austria).

### Ethics and trial registration

This study was approved by the Institutional Review Board (IRB) of Asan Medical Center (IRB No. 2017 − 0315; approved on 9 March 2017). Written informed consent was obtained from both legal guardians prior to enrollment in the prospective randomized trial. The trial was registered with the Korean Clinical Trials Registry (KCT0002373; registered 7 July 2017).

## Results

### Cohort results

**Fig. 1:**
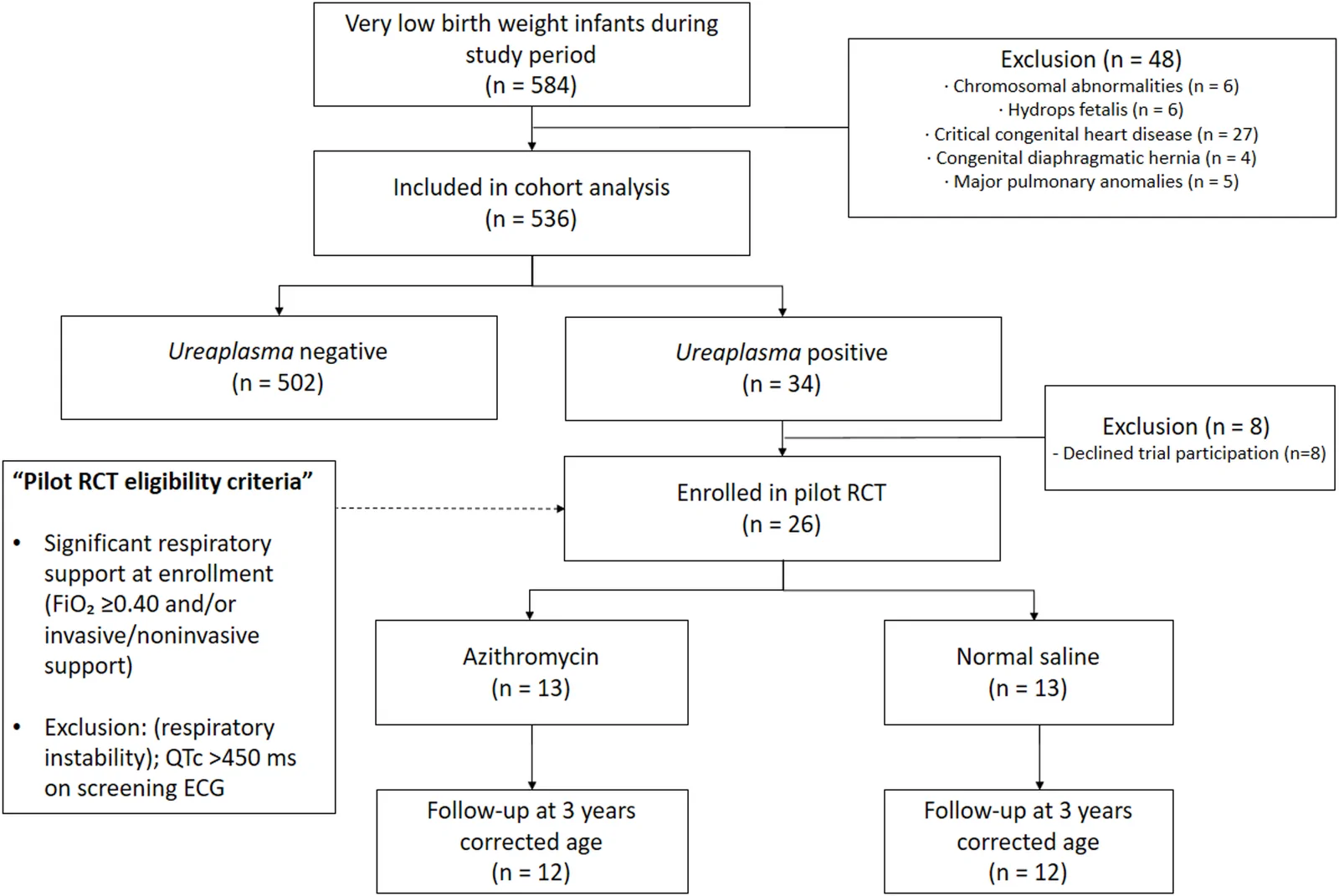
Study population flow diagram

Among 536 eligible VLBW infants in the cohort, 34 (6.3%) were *Ureaplasma* positive and 502 were *Ureaplasma* negative (Fig. 1). All infants underwent paired screening with one respiratory specimen (nasopharyngeal or endotracheal, depending on intubation status) and one gastric aspirate culture. *Ureaplasma* was detected in the gastric aspirate only in 4 infants, in the respiratory specimen only in 4 infants, and in both specimens in 26 infants. Accordingly, the positivity rate was 5.6% (30/536) for gastric aspirates and 5.6% (30/536) for respiratory specimens. *Ureaplasma*-positive infants were born at a lower gestational age than *Ureaplasma*-negative infants (27.2 ± 1.8 vs. 29.1 ± 3.4 weeks; *P* = 0.002). Histologic chorioamnionitis and PPROM were more frequent in the *Ureaplasma*-positive group (23/33 [69.7%] vs. 154/499 [30.9%], *P* < 0.001; and 21/34 [61.8%] vs. 130/502 [25.9%], *P* < 0.001, respectively) (Table 1). Baseline characteristics according to respiratory-specimen positivity (respiratory *Ureaplasma*) are summarized in Supplementary Table S1. This table describes the respiratory-positive subset used in the respiratory-specimen-restricted sensitivity analyses.

**Table 1:** Comparison of demographics and clinical characteristics according to *Ureaplasma*

	Ureaplasma (+) (*n* = 34)	Ureaplasma (-) (*n* = 502)	*P*-value
Gestational age (weeks)	27.2 (1.8)	29.1 (3.4)	0.002
Birth weight (g)	972.9 (303.5)	1,018.3 (299.7)	0.393
Male, n (%)	19 (55.9)	255 (50.8)	0.558
Multiplicity, n (%)	10 (29.4)	287 (57.2)	0.002
Maternal hypertension, n (%)	1 (2.9)	115 (22.9)	0.006
Maternal diabetes, n (%)	3 (8.8)	55 (11.0)	0.698
Histologic chorioamnionitis, n/N (%)	23/33 (69.7)	154/499 (30.9)	< 0.001
PPROM, n (%)	21 (61.8)	130 (25.9)	<0.001
Antenatal corticosteroid, n (%)	34 (100)	483 (96.2)	0.624
Cesarean section, n (%)	22 (64.7)	417 (83.1)	0.007
Respiratory distress syndrome, n (%)	30 (88.2)	342 (68.1)	0.014
Air leak syndrome, n (%)	2 (5.9)	12 (2.4)	0.221
Medical or surgical treatment of PDA, n (%)	17 (50)	176 (35.1)	0.079
NEC (stage ≥ 2), n (%)	3 (8.8)	31 (6.2)	0.468
Late-onset sepsis, n (%)	15 (44.1)	137 (27.3)	0.035
IVH (grade ≥ 3), n/N (%)	3/33 (9.1)	31/454 (6.8)	0.494
ROP (stage ≥ 3), n/N (%)	11/33 (33.3)	63/441 (14.3)	0.004
Mortality, n (%)	4 (11.8)	35 (7.0)	0.298
Moderate-to-severe BPD, n/N (%)	26/34 (76.5)	188/458 (41.0)	< 0.001
Severe BPD, n/N (%)	23/34 (67.6)	165/458 (36.0)	< 0.001
Systemic corticosteroid for BPD, n (%)	19 (55.9)	120 (23.9)	< 0.001
Home oxygen therapy, n/N (%)	12/26 (46.2)	81/424 (19.1)	0.002

Continuous variables are expressed as means ± standard deviations. *PPROM* preterm premature rupture of membranes, *PDA* patent ductus arteriosus, *NEC* necrotizing enterocolitis, *IVH* intraventricular hemorrhage, *ROP* retinopathy of prematurity, *BPD* bronchopulmonary dysplasia

Data are presented as n (%) unless otherwise specified; when data were missing, values are shown as n/N (%), where N reflects available data

BPD outcomes were assessed at 36 weeks’ postmenstrual age among infants who survived to 36 weeks and had documented 36-week respiratory status

Home oxygen therapy was assessed among infants with available outpatient follow-up records

**Fig. 2:**
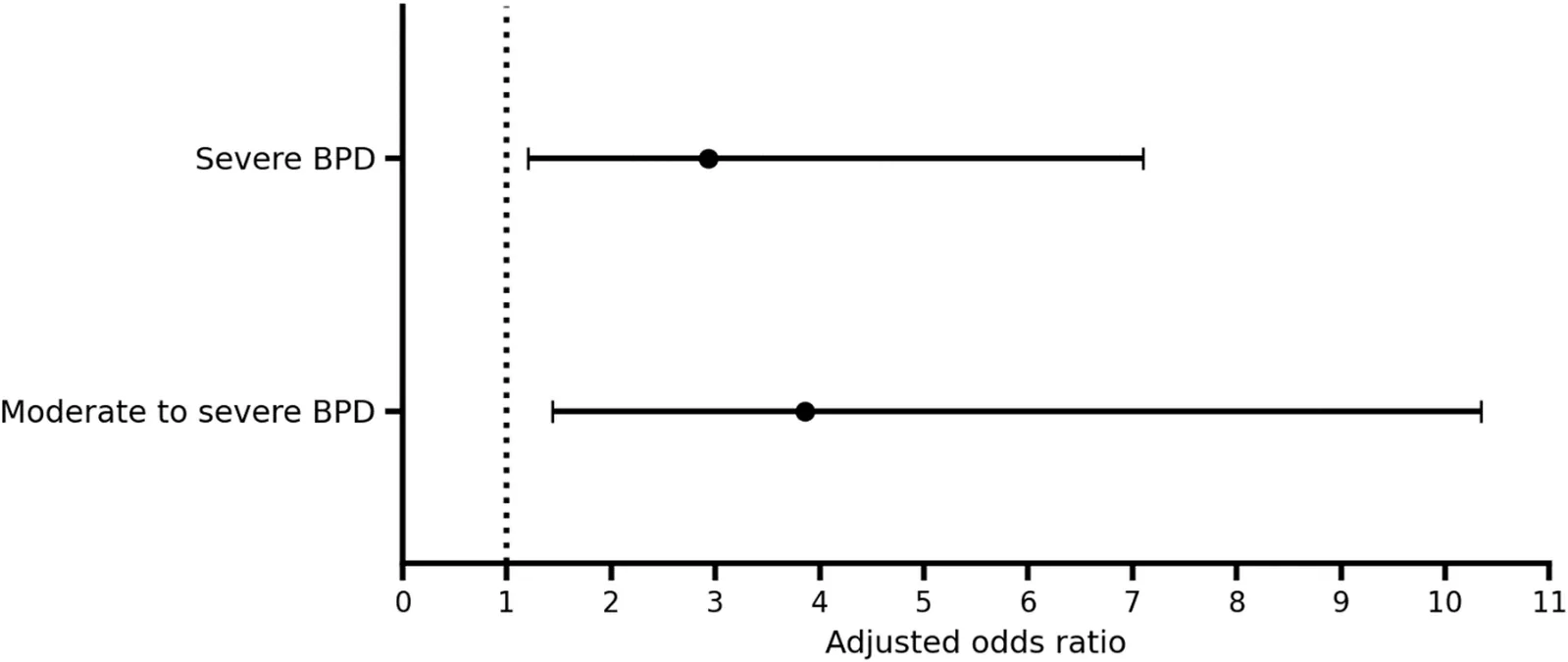
Adjusted odds ratios (aORs) for moderate-to-severe bronchopulmonary dysplasia (BPD) and severe BPD associated with Ureaplasma positivity. Estimates were obtained from multivariable logistic regression models adjusted for gestational age, sex, preterm premature rupture of membranes, histologic chorioamnionitis, antenatal steroid exposure, patent ductus arteriosus treatment, and sepsis. Points indicate aORs and horizontal lines represent 95% confidence intervals

Respiratory outcomes at 36 weeks’ postmenstrual age differed substantially between groups. Moderate-to-severe BPD occurred more frequently among *Ureaplasma*-positive infants than among *Ureaplasma*-negative infants (26/34 [76.5%] vs. 188/458 [41.0%]; *P* < 0.001), and severe BPD showed a similar pattern (23/34 [67.6%] vs. 165/458 [36.0%]; *P* < 0.001). Home oxygen therapy after discharge was also more common in the *Ureaplasma*-positive group (12/26 [46.2%] vs. 81/424 [19.1%]; *P* = 0.002) (Table 1). After adjustment for gestational age, sex, PPROM, histologic chorioamnionitis, antenatal steroid exposure, PDA treatment, and sepsis, *Ureaplasma* positivity remained associated with higher odds of moderate-to-severe BPD (aOR 3.86, 95% CI 1.44–10.36) and severe BPD (aOR 2.93, 95% CI 1.21–7.10) (Fig. 2). In sensitivity analyses restricted to infants with respiratory-specimen positivity, the adjusted associations with moderate-to-severe and severe BPD remained directionally consistent with the main analysis (Supplementary Table S2).

### Pilot trial results

Twenty-six *Ureaplasma*-positive infants were enrolled and randomized (13 azithromycin and 13 placebo). Baseline maternal and neonatal characteristics were generally comparable between groups (Table 2). Among trial participants, *Ureaplasma* positivity was detected from gastric aspirate alone in 3 (12%), respiratory aspirate alone in 2 (8%; endotracheal or nasopharyngeal based on intubation status), or multiple sites in 21 (81%) (Supplementary Table S3). Azithromycin was initiated at a median of 5 days of life (IQR, 3.5–6). Species identification was available for trial participants: all 26 infants were identified as *Ureaplasma urealyticum* (0 *U. parvum*). *Mycoplasma hominis* co-detection was observed in 8 of 26 infants.

**Table 2:** Baseline characteristics and key clinical outcomes in the double-blind, placebo-controlled pilot trial of azithromycin

	Azithromycin (*n* = 13)	Normal saline (*n* = 13)	*P*-value
Gestational age, weeks (SD)	27.0 (1.6)	27.2 (1.9)	0.774
Birth weight, g (SD)	988.5 (221.0)	993.7 (306.8)	0.961
Male, n (%)	7 (53.8)	8 (61.5)	1.000
Multiplicity, n (%)	5 (38.5)	3 (23.1)	0.673
Maternal diabetes, n (%)	2 (15.4)	1 (7.7)	1.000
Histologic chorioamnionitis, n/N (%)	10/13 (76.9)	10/12 (83.3)	1.000
PPROM, n (%)	8 (61.5)	9 (69.2)	1.000
Antenatal corticosteroid, n (%)	13 (100)	13 (100)	1.000
Cesarean section, n (%)	8 (61.5)	7 (53.8)	1.000
Respiratory distress syndrome, n (%)	12 (92.3)	11 (84.6)	1.000
Air leak syndrome, n (%)	0 (0.0)	0 (0.0)	—
Medical or surgical treatment of PDA, n (%)	8 (61.5)	5 (38.5)	0.434
NEC (stage ≥ 2), n (%)	0 (0.0)	2 (15.4)	0.480
Late-onset sepsis, n (%)	6 (46.2)	5 (38.5)	1.000
IVH (grade ≥ 3), n (%)	1 (7.7)	2 (15.4)	1.000
ROP (stage ≥ 3), n (%)	3 (23.1)	4 (30.8)	1.000
Mortality, n (%)	1 (7.7)	1 (7.7)	1.000

Continuous variables are expressed as means ± standard deviations. *PPROM* preterm premature rupture of membranes, *PDA* patent ductus arteriosus, *NEC* necrotizing enterocolitis, *IVH* intraventricular hemorrhage, *ROP* retinopathy of prematurity

Data are presented as n (%) unless otherwise specified; when data were missing, values are shown as n/N (%), where N reflects available data. Between-group comparisons were performed using the Mann–Whitney U test for continuous variables and Fisher’s exact test for categorical variables. P-values are exploratory given the pilot sample size. P-value was not calculated when there were no events in either group

Point estimates favored azithromycin for the primary outcomes of moderate-to-severe BPD [61.5% (8/13) vs. 84.6% (11/13); RR 0.73, 95% CI 0.45–1.19; RD -23.1%, 95% CI -60.2 to 24.5] and severe BPD [46.2% (6/13) vs. 76.9% (10/13); RR 0.60, 95% CI 0.31–1.16; RD -30.8%, 95% CI -68.6 to 21.1], although confidence intervals crossed 1 given the small pilot sample size (Table 3). Given the pilot sample size, confidence intervals were wide and the trial was not powered for definitive clinical endpoints; therefore, these estimates should be interpreted as hypothesis-generating.

**Table 3:** Outcomes of azithromycin versus placebo in *Ureaplasma*-positive very low birth weight infants: respiratory and exploratory 3-year neurodevelopmental outcomes

	Azithromycin (*n* = 13)	Normal saline (*n* = 13)	*P*-value	RD, % points (95% CI)	RR (95% CI)
Respiratory outcome					
Moderate-to-severe BPD, n (%)	8 (61.5)	11 (84.6)	0.185	-23.1 (-60.2, + 24.5)	0.73 (0.45–1.19)
Severe BPD, n (%)	6 (46.2)	10 (76.9)	0.107	-30.8 (-68.6, + 21.1)	0.60 (0.31–1.16)
Systemic corticosteroid for BPD, n (%)	7 (53.8)	5 (38.5)	0.431	+ 15.4 (-35.3, + 59.1)	1.40 (0.60–3.28)
Duration of invasive mechanical ventilation, days (IQR)	16 (0–41.5)	20 (2.5–43.5)	0.886		
Duration of noninvasive respiratory support, days (IQR)	50 (36.5–53.0)	54 (37.5–70.5)	0.312		
Home oxygen therapy, n (%)	3/11 (27.3)	6/11 (54.5)	0.387	-27.3 (-69.0, + 28.6)	0.50 (0.17–1.51)
Exploratory neurodevelopmental outcome at 3 years of age					
Hearing impairment, n/N (%)	2/12 (16.7)	1/12 (8.3)	0.303		
Visual impairment, n/N (%)	0/10 (0)	0/11 (0)	NA		
Cerebral palsy, n/N (%)	1/10 (10)	1/11 (9.1)	0.943		
Severe mental delay (BSID-II MDI < 70), n/N (%)	2/5 (40)	1/8 (12.5)	0.315		
Severe motor delay (BSID-II PDI < 70), n/N (%)	2/5 (40)	2/8 (25)	0.510		

*BPD* bronchopulmonary dysplasia, *IQR* interquartile range, *BSID-II* Bayley Scales of Infant Development, Second Edition, *MDI* mental developmental index, *PDI* psychomotor developmental index

Data are presented as n (%) unless otherwise specified; when data were missing, values are shown as n/N (%), where N reflects available data. Continuous variables are expressed as median with interquartile range. Risk differences (95% CIs) were calculated using the Newcombe method with Wilson score intervals; risk ratios (95% CIs) were calculated on the log scale. P values for categorical variables were obtained using Fisher’s exact test, and continuous variables were compared using the Mann-Whitney U test

Home oxygen therapy was assessed among infants with available outpatient follow-up records

### Microbiologic response, safety, and exploratory neurodevelopmental outcomes

Microbiologic response supported biological activity of the regimen: loss of detectable *Ureaplasma* by culture was observed in 13/13 infants in the azithromycin group, whereas persistent *Ureaplasma* positivity at 2 weeks was observed in 5/13 infants in the placebo group. Because follow-up specimens could differ by intubation status, follow-up results were interpreted as loss of detectable *Ureaplasma* by culture rather than definitive lower-airway eradication.

No QTc prolongation was observed on follow-up electrocardiography, and no clinically significant safety concerns attributable to azithromycin were identified. Overall, no safety signal was identified with the two-week azithromycin regimen in this pilot trial.

Exploratory neurodevelopmental outcomes at 3 years of corrected age were available for a subset of infants and included BSID-II severe delay (MDI or PDI < 70), cerebral palsy, hearing impairment, and visual impairment; these outcomes are presented descriptively given incomplete follow-up (Table 3).

## Discussion

In this single-center study of VLBW infants, *Ureaplasma* positivity was associated with substantially worse respiratory outcomes, including higher rates of moderate-to-severe BPD, severe BPD, and home oxygen therapy. In a nested double-blind pilot randomized trial among *Ureaplasma*-positive infants, a two-week intravenous azithromycin regimen initiated early in postnatal life showed directionally favorable estimates for both primary BPD outcomes compared with placebo, although confidence intervals were wide given the pilot sample size. Microbiologic response supported biological activity of the regimen, with loss of detectable *Ureaplasma* by culture in all treated infants, and no QTc prolongation or clinically significant safety concerns were identified. Taken together, these data identify *Ureaplasma* positivity as a clinically important high-risk subgroup for severe BPD and provide preliminary randomized and microbiologic signals to inform future adequately powered, microbiology-guided trials [17, 18].

Our cohort findings support that *Ureaplasma* is associated with clinically meaningful respiratory morbidity in VLBW infants. Even after adjustment for major perinatal confounders, *Ureaplasma* positivity remained associated with increased odds of both moderate-to-severe and severe BPD, consistent with prior systematic reviews and meta-analyses [6–9]. The association with home oxygen therapy after discharge further suggests that early *Ureaplasma* exposure may contribute to persistent respiratory burden beyond the neonatal hospitalization, a patient-centered outcome with practical implications for families and follow-up care [1, 2].

The observed cohort signal is biologically plausible and supported by experimental and translational studies describing *Ureaplasma* as a trigger of sustained pulmonary inflammation in the developing lung. Antenatal or perinatal exposure can induce cytokine and chemokine upregulation, recruit inflammatory cells, and promote remodeling that may impair alveolar and pulmonary vascular development [19–22]. These effects may be amplified in extremely preterm infants, in whom immature innate immune responses can be dysregulated and may prime the lung for exaggerated injury with subsequent exposures such as mechanical ventilation, oxygen, or secondary infection [4–5, 21, 23]. Mechanistic work also supports activation of pattern-recognition pathways by *Ureaplasma* components, providing a plausible upstream link to persistent inflammatory signaling in the preterm lung [21, 23]. Within this framework, *Ureaplasma* may function as a disease modifier that increases susceptibility to more severe BPD phenotypes rather than acting as a sole determinant [1, 4, 5].

Our observational cohort analysis supports an association between culture-detectable *Ureaplasma* and adverse respiratory outcomes, whereas the nested randomized pilot trial provides only preliminary, imprecise estimates of treatment effect due to limited sample size. Accordingly, the randomized findings should be viewed as a feasibility and signal-generating result that warrants confirmation in adequately powered trials. Given the small sample size, the consistent direction of effect estimates across both primary BPD outcomes should be viewed alongside the wide confidence intervals, which remain compatible with both clinically meaningful benefit and no effect [17, 18]. This approach aligns with guidance for pilot and feasibility trials that emphasizes estimation to inform the design of adequately powered confirmatory studies [17, 18]. Therefore, our pilot results primarily provide preliminary effect-size estimates, feasibility information, and complementary biological evidence to guide future work. Consistent with CONSORT guidance for pilot trials, these estimates are intended to inform effect sizes and sample size planning for future confirmatory studies rather than to establish efficacy [18].

Interpretation of our pilot findings should also consider heterogeneity in the macrolide literature. Clarithromycin has been reported to reduce BPD among *Ureaplasma* urealyticum-positive preterm infants, supporting a targeted treatment concept in a microbiologically defined subgroup [10]. In contrast, broader prophylactic approaches in unselected very preterm populations have yielded mixed results, and the AZTEC trial did not demonstrate improvement in its primary endpoint with early intravenous azithromycin [12]. Differences across studies likely relate to variation in patient selection, specimen source and ascertainment of lower-airway involvement, timing of treatment initiation, and dosing strategies [11, 12].

Treatment timing may be an important determinant of the clinical effects of macrolide therapy in this setting. In our nested pilot trial, the median initiation at day 5 reflects a pragmatic, microbiology-guided approach, because the assigned study treatment could be started only after culture confirmation of Ureaplasma positivity. Clinical stabilization and completion of written parental consent could also contribute to variability in initiation timing. In this context, ‘early’ in our study refers to initiation as soon as feasible after microbiologic confirmation, rather than routine prophylaxis within the first 24–48 h after birth. This differs from prior trials that were designed to start macrolide therapy earlier in postnatal life. For instance, in the clarithromycin trial by Ozdemir et al., screening swabs were obtained within the first 3 postnatal days and Ureaplasma-positive infants were randomized thereafter, aiming to initiate treatment soon after microbiologic screening [10]. In contrast, broader or prophylactic azithromycin strategies in unselected preterm populations have shown inconsistent effects on respiratory outcomes, and the AZTEC trial did not meet its primary endpoint despite an early intravenous azithromycin strategy [12]. Pharmacokinetic and microbial-response data also suggest that achieving adequate exposure early in life may be relevant for microbiologic response, although neonatal trials have not directly compared different initiation timepoints [11]. Therefore, while earlier initiation could plausibly enhance antimicrobial and immunomodulatory effects, the clinical impact of starting on day 5 versus within the first few postnatal days remains uncertain. Future trials would benefit from faster diagnostics (e.g., rapid molecular testing) to shorten the interval between screening and treatment initiation while preserving a targeted design.

Our two-week step-down regimen (10 mg/kg/day for one week followed by 5 mg/kg/day for one week) was selected to balance sustained antimicrobial exposure with tolerability in a fragile population. Neonatal azithromycin regimens reported in studies published after our study was initiated have included shorter courses, such as a 10-day early intravenous regimen in the AZTEC trial protocol, providing context for comparability with more recent trials [12]. A potential advantage is feasibility and potentially favorable safety, while maintaining exposure that may be sufficient for organism suppression. A potential limitation is that compared with higher-dose regimens, it may not reliably achieve lower-airway eradication in all infants, particularly when organism burden is high or lower-airway involvement is established early [11, 12]. In our study, loss of detectable *Ureaplasma* by culture was observed in all treated infants, whereas persistence at two weeks was observed in a substantial subset of controls, supporting biological activity. However, follow-up specimens at two weeks could differ by intubation status, and upper-airway or gastric specimens may not fully represent lower-airway colonization [6, 7]. Therefore, microbiologic outcomes should be interpreted conservatively as loss of detectable organism under the study sampling strategy rather than definitive lower-airway eradication. Gastric aspirates have been reported to reflect antenatal *Ureaplasma* exposure and, in some studies, to correlate with respiratory tract colonization in VLBW infants, supporting its use as a pragmatic screening tool in routine neonatal care [9].

If azithromycin ultimately reduces BPD risk, two mechanisms are plausible: antimicrobial activity and immunomodulation. Macrolides can modulate inflammatory pathways independent of bacterial killing, which may be relevant to the inflammatory milieu of evolving BPD [4, 5]. Because we did not measure inflammatory biomarkers, the contribution of immunomodulation cannot be assessed in this study. Nonetheless, the combination of microbiologic response and directionally favorable clinical estimates provides a coherent rationale for further evaluation while avoiding causal claims [11, 17, 18].

Safety remains central for macrolide use in preterm infants. QT prolongation and rare arrhythmias have been reported with macrolides, typically in the presence of additional risk factors or interacting medications [24]. Early-life macrolide exposure has also been associated with infantile hypertrophic pyloric stenosis in epidemiologic studies, underscoring the importance of surveillance in larger trials [25]. In our pilot trial, no QTc prolongation was observed on follow-up electrocardiography, and no clinically significant safety concerns attributable to azithromycin were identified. Overall, no safety signal was identified with the two-week azithromycin regimen in this pilot trial. While these findings are reassuring, the sample size precludes exclusion of rare adverse events, and confirmatory studies should incorporate protocolized safety monitoring [18, 24].

This study has limitations. The cohort analysis is observational and susceptible to residual confounding. The pilot randomized trial was small and not powered for efficacy, resulting in wide confidence intervals [17, 18]. Microbiologic follow-up was limited by specimen-type variability, and PCR-based quantification and inflammatory biomarkers were not available. Detection relied on culture-based methods, which may be less sensitive than PCR-based assays. *Ureaplasma* colonization may have been underestimated, and any nondifferential misclassification could have biased associations toward the null. Neurodevelopmental outcomes were exploratory with incomplete follow-up and should be interpreted descriptively. Loss to follow-up may have been nonrandom, as infants with greater medical complexity and families facing logistical or socioeconomic barriers may be less likely to return for standardized testing, potentially biasing between-group comparisons in either direction. Therefore, these neurodevelopmental findings should be interpreted cautiously and are presented for descriptive purposes only. We used the 2001 NICHD consensus definition to classify BPD severity because the cohort and trial were designed and initiated in 2017, prior to publication and broader adoption of contemporary definitions such as the Jensen classification [2]. Differences in BPD definitions may affect direct comparability with more recent studies. Although we did not reclassify outcomes post hoc, applying a support-based definition (e.g., Jensen) could shift the distribution of BPD severity, particularly among infants managed with noninvasive support at 36 weeks’ PMA, and the overall direction of the main findings may remain broadly similar. Importantly, our microbiologic endpoint represents loss of detectable *Ureaplasma* by culture rather than definitive lower-airway eradication. This should be interpreted cautiously, as gastric aspirates may reflect antenatal exposure or swallowed amniotic fluid rather than true lower-airway colonization, and specimen source may differ by intubation status and sampling strategy.

Despite these limitations, the study has important strengths. The combined cohort and nested double-blind randomized pilot design provides complementary evidence: the cohort demonstrates a clinically meaningful association between *Ureaplasma* positivity and severe respiratory morbidity, while the pilot trial adds randomized, blinded estimates of effect direction together with microbiologic response and structured safety assessment [17, 18]. Inclusion of home oxygen therapy complements 36-week BPD severity and enhances clinical relevance [1, 2].

## Conclusion

In conclusion, *Ureaplasma* positivity in VLBW infants was associated with markedly worse respiratory outcomes, including severe BPD and the need for home oxygen therapy. In a nested double-blind pilot trial among *Ureaplasma*-positive infants, a two-week intravenous azithromycin regimen achieved loss of detectable organism and showed directionally favorable estimates for both moderate-to-severe and severe BPD without an observed safety signal. These findings support feasibility and biological activity of targeted therapy and justify adequately powered trials with standardized microbiologic ascertainment and rigorous safety monitoring.

## Supplementary Information

Supplementary Material 1.

## Data Availability

The datasets used or analyzed during the current study are available from the corresponding author on reasonable request.

## Abbreviations

aOR: adjusted odds ratio
BPD: bronchopulmonary dysplasia
BSID-II: Bayley Scales of Infant Development, Second Edition
CI: confidence interval
FiO₂: fraction of inspired oxygen
GA: gestational age
HFOV: high-frequency oscillatory ventilation
IVH: intraventricular hemorrhage
MDI: mental developmental index
NEC: necrotizing enterocolitis
NICHD: National Institute of Child Health and Human Development
NICU: neonatal intensive care unit
PDA: patent ductus arteriosus
PDI: psychomotor developmental index
PPROM: preterm premature rupture of membranes
QTc: corrected QT interval
RDS: respiratory distress syndrome
ROP: retinopathy of prematurity
RR: risk ratio
SD: standard deviation
VLBW: very low birth weight

## References

[1] Thébaud B, Goss KN, Laughon M, Whitsett JA, Abman SH, Steinhorn RH, et al. Bronchopulmonary dysplasia. Nat Rev Dis Primers. 2019;5(1):78. 10.1038/s41572-019-0127-7. PMC6986462 31727986

[2] Jensen EA, Dysart K, Gantz MG, McDonald S, Bamat NA, Keszler M, et al. The diagnosis of bronchopulmonary dysplasia in very preterm infants: an evidence-based approach. Am J Respir Crit Care Med. 2019;200(6):751–9. 10.1164/rccm.201812-2348OC. PMC6775872 30995069

[3] Jobe AH, Bancalari E. Bronchopulmonary dysplasia. Am J Respir Crit Care Med. 2001;163(7):1723–9. 10.1164/ajrccm.163.7.2011060. 11401896

[4] Speer CP. Inflammation and bronchopulmonary dysplasia. Semin Neonatol. 2003;8(1):29–38. 10.1016/S1084-2756(02)00190-2. 12667828

[5] Balany J, Bhandari V. Understanding the impact of infection, inflammation, and their persistence in the pathogenesis of bronchopulmonary dysplasia. Front Med (Lausanne). 2015;2:90. 10.3389/fmed.2015.00090. PMC4685088 26734611

[6] Gwee A, Curtis N. Ureaplasma – Are you sitting comfortably? J Infect. 2014;68(Suppl 1):S19–23. 10.1016/j.jinf.2013.09.027. 24139189

[7] Viscardi RM, Kallapur SG. Role of Ureaplasma respiratory tract colonization in bronchopulmonary dysplasia pathogenesis: current concepts and update. Clin Perinatol. 2015;42(4):719–38. 10.1016/j.clp.2015.08.003. PMC4662049 26593075

[8] Lowe J, Watkins WJ, Edwards MO, Spiller OB, Jacqz-Aigrain E, Kotecha SJ, Kotecha S. Association between pulmonary ureaplasma colonization and bronchopulmonary dysplasia in preterm infants: updated systematic review and meta-analysis. Pediatr Infect Dis J. 2014;33(7):697–702. 10.1097/INF.0000000000000239. 24445836

[9] Chen Y, Zhang Y, Zhang Y, Yang D, Wang X, Wang Y, et al. Association between Ureaplasma urealyticum colonization and bronchopulmonary dysplasia in preterm infants: a systematic review and meta-analysis. Front Pediatr. 2024;12:1436568. 10.3389/fped.2024.1436568. PMC11338929 39175806

[10] Ozdemir R, Erdeve O, Dizdar EA, Oguz SS, Uras N, Saygan S, et al. Clarithromycin in preventing bronchopulmonary dysplasia in Ureaplasma urealyticum-positive preterm infants. Pediatrics. 2011;128(6):e1496–501. 10.1542/peds.2011-1350. 22123897

[11] Viscardi RM, Othman AA, Hassan HE, Eddington ND, Abebe E, Terrin ML, Kaufman DA, Waites KB. Azithromycin to prevent bronchopulmonary dysplasia in ureaplasma-infected preterm infants: pharmacokinetics, safety, microbial response, and clinical outcomes with a 20-milligram-per-kilogram single intravenous dose. Antimicrob Agents Chemother. 2013;57(5):2127–33. 10.1128/AAC.02183-12. PMC3632913 23439637

[12] Lowe J, Gillespie D, Aboklaish A, Lau TMM, Consoli C, Babu M, et al. Azithromycin therapy for prevention of chronic lung disease of prematurity (AZTEC): a multicentre, double-blind, randomised, placebo-controlled trial. Lancet Respir Med. 2024;12(8):608–18. 10.1016/S2213-2600(24)00079-1. 38679042

[13] Laughon MM, Langer JC, Bose CL, et al. Prediction of bronchopulmonary dysplasia by postnatal age in extremely premature infants. Am J Respir Crit Care Med. 2011;183(12):1715–22. 10.1164/rccm.201101-0055OC. PMC3136997 21471086

[14] Bell MJ, Ternberg JL, Feigin RD, et al. Neonatal necrotizing enterocolitis. Therapeutic decisions based upon clinical staging. Ann Surg. 1978;187(1):1–7. 10.1097/00000658-197801000-00001. PMC1396409 413500

[15] Papile LA, Burstein J, Burstein R, Koffler H. Incidence and evolution of subependymal and intraventricular hemorrhage: a study of infants with birth weights less than 1,500 gm. J Pediatr. 1978;92(4):529–34. 10.1016/S0022-3476(78)80282-0. 305471

[16] International Committee for the Classification of Retinopathy of Prematurity. The International Classification of Retinopathy of Prematurity revisited. Arch Ophthalmol. 2005;123(7):991–9. 10.1001/archopht.123.7.991. 16009843

[17] Lee EC, Whitehead AL, Jacques RM, Julious SA. The statistical interpretation of pilot trials: should significance thresholds be reconsidered? Pharm Stat. 2014;13(6):313–23. 10.1002/pst.1646. PMC3994566 24650044

[18] Eldridge SM, Chan CL, Campbell MJ, Bond CM, Hopewell S, Thabane L, et al. CONSORT 2010 statement: extension to randomised pilot and feasibility trials. BMJ. 2016;355:i5239. 10.1136/bmj.i5239. PMC5076380 27777223

[19] Collins JJP, Kallapur SG, Knox CL, Nitsos I, Polglase GR, Pillow JJ, et al. Inflammation in fetal sheep from intra-amniotic injection of Ureaplasma parvum. Am J Physiol Lung Cell Mol Physiol. 2010;299(6):L852–60. 10.1152/ajplung.00311.2010. PMC3006269 20935228

[20] Moss TJM, Nitsos I, Ikegami M, Jobe AH, Newnham JP. Experimental intrauterine Ureaplasma infection in sheep. Am J Obstet Gynecol. 2005;192(4):1179–86. 10.1016/j.ajog.2004.11.063. 15846199

[21] Kallapur SG, Kramer BW, Knox CL, Berry CA, Collins JJP, Kemp MW, et al. Chronic fetal exposure to Ureaplasma parvum suppresses innate immune responses in sheep. J Immunol. 2011;187(5):2688–95. 10.4049/jimmunol.1100779. PMC3159703 21784974

[22] Willems MGM, Kemp MW, Fast LA, Wagemaker NMM, Janssen LEW, Newnham JP, et al. Pulmonary vascular changes in extremely preterm sheep after intra-amniotic exposure to Ureaplasma parvum and lipopolysaccharide. PLoS ONE. 2017;12(6):e0180114. 10.1371/journal.pone.0180114. PMC5493356 28666032

[23] Viscardi RM, Manimtim WM, Sun CC, Duffy L, Cassell GH. Ureaplasma urealyticum modulates endotoxin-induced cytokine responses in monocytes from preterm and term newborn infants and adults. Infect Immun. 2001;69(6):3906–15. 10.1128/IAI.69.6.3906-3915.2001. PMC98421 11349058

[24] Hancox JC, Hasnain M, Vieweg WVR, Crouse EL, Baranchuk A. Azithromycin, cardiovascular risks, QTc interval prolongation and torsade de pointes: narrative review. Ther Adv Infect Dis. 2013;1(5):155–65. 10.1177/2049936113501816. PMC4040726 25165550

[25] Eberly MD, Eide MB, Thompson JL, Nylund CM. Azithromycin in early infancy and pyloric stenosis. Pediatrics. 2015;135(3):483–8. 10.1542/peds.2014-2027. PMC9923582 25687145

