# Supplementary material for: Ureaplasma positivity and respiratory outcomes in very low birth weight infants: a cohort study with a nested double-blind randomized pilot trial of azithromycin
Source: BMC Pediatr. 2026 Jul 3;26:955. doi: 10.1186/s12887-026-07199-2 (PMC13640359; doi:10.1186/s12887-026-07199-2)
Supplement: Supplementary file 1 [file 12887_2026_7199_MOESM1_ESM.docx]

**Supplementary Table S1. Baseline characteristics according to respiratory-specimen positivity (Respiratory *Ureaplasma*)**

Respiratory *Ureaplasma*(+) was defined as culture positivity from endotracheal or nasopharyngeal aspirates (with or without concomitant gastric aspirate positivity). Infants with gastric-aspirate-only positivity (n=4) were excluded.

| **Characteristic** | **Respiratory *Ureaplasma* (+) (n = 30)** | ***Ureaplasma* (–) (n = 502)** | **P-value** |
| --- | --- | --- | --- |
| Gestational age (weeks) | 27.6 (1.6) | 29.1 (3.4) | 0.003 |
| Birth weight (g) | 990.0 (252.0) | 1,018.3 (299.7) | 0.529 |
| Male, n (%) | 15 (50.0) | 255 (50.8) | 1.000 |
| Multiplicity, n (%) | 9 (30.0) | 287 (57.2) | 0.011 |
| Maternal hypertension, n (%) | 1 (3.3) | 115 (22.9) | 0.015 |
| Maternal diabetes, n (%) | 3 (10.0) | 55 (11.0) | 1.000 |
| Histologic chorioamnionitis, n/N (%) | 23/29 (79.3) | 154/499 (30.9) | <0.001 |
| PPROM, n (%) | 18 (60.0) | 130 (25.9) | <0.001 |
| Antenatal corticosteroid, n (%) | 30 (100.0) | 483 (96.2) | 0.389 |
| Cesarean section, n (%) | 16 (53.3) | 417 (83.1) | 0.008 |
| Respiratory distress syndrome, n (%) | 24 (80.0) | 342 (68.1) | 0.009 |
| Air leak syndrome, n (%) | 3 (10.0) | 12 (2.4) | 0.110 |
| Medical or surgical treatment of PDA, n (%) | 15 (50.0) | 176 (35.1) | 0.052 |
| NEC (stage ≥2), n (%) | 3 (10.0) | 31 (6.2) | 0.702 |
| Late-onset sepsis, n (%) | 9 (30.0) | 137 (27.3) | 0.052 |
| IVH (grade ≥3), n/N (%) | 3/28 (10.7) | 31/454 (6.8) | 0.565 |
| ROP (stage ≥3), n/N (%) | 10/28 (35.7) | 63/441 (14.3) | 0.036 |
| Mortality, n (%) | 4 (13.3) | 35 (7.0) | 0.149 |
| Moderate to severe BPD, n/N (%) | 20/28 (71.4) | 188/458 (41.0) | <0.001 |
| Severe BPD, n/N (%) | 18/28 (64.3) | 165/458 (36.0) | <0.001 |
| Systemic corticosteroid for BPD, n (%) | 16 (53.3) | 120 (23.9) | <0.001 |
| Home oxygen therapy, n/N (%) | 10/28 (35.7) | 81/424 (19.1) | 0.015 |

Values are mean (SD) for continuous variables and n/N (%) for categorical variables. P-values were calculated using the Mann–Whitney U test for continuous variables and Fisher’s exact test or chi-square test for categorical variables, as appropriate.

**Supplementary Table S2. Sensitivity analyses restricted to respiratory-specimen positivity**

| Outcome | Main model  (Ureaplasma any-site +)  aOR (95% CI) | Sensitivity  (Respiratory + only)  aOR (95% CI) |
| --- | --- | --- |
| Moderate-to-severe BPD | 3.86 (1.44–10.35) | 2.51 (1.00–6.29) |
| Severe BPD | 2.93 (1.21–7.10) | 2.59 (1.09–6.18) |

Estimates were obtained from multivariable logistic regression models adjusted for gestational age, sex, preterm premature rupture of membranes, histologic chorioamnionitis, antenatal steroid exposure, PDA treatment, and sepsis. Sensitivity analyses excluded infants with gastric-aspirate-only positivity and defined respiratory-specimen positivity as culture positivity from endotracheal or nasopharyngeal aspirates (with or without concomitant gastric aspirate positivity).

**Supplementary Table S3. Patterns of Ureaplasma detection by specimen source among trial participants (n=26)**

| **Pattern** | **n (%)** |
| --- | --- |
| Gastric aspirate only | 3 (11.5) |
| Respiratory specimen only | 2 (7.7) |
| Multiple sites (gastric and respiratory) | 21 (80.8) |

Respiratory specimens included endotracheal aspirates (intubated infants) or nasopharyngeal aspirates (non-intubated infants). Detection was culture-based.
